# Modification of oncoplastic breast surgery with immediate volume replacement using a thoracodorsal adipofascial flap

**DOI:** 10.1007/s12282-022-01331-7

**Published:** 2022-02-04

**Authors:** Munetsugu Hirata, Hiroko Toda, Naotomo Higo, Yoshiaki Shinden, Takao Ohtsuka, Yuko Kijima

**Affiliations:** 1 Department of Breast Surgery, Fujita Health University, School of Medicine, 1-98 Dengakugakubo, Kutsukake-cho, Toyoake, Aichi 470-1192 Japan; 2 Department of Digestive Surgery, Breast and Thyroid Surgery, Kagoshima University Graduate School of Medical and Dental Sciences, 8-35-1 Sakuragaoka, Kagoshima, 890-8520 Japan

**Keywords:** Breast cancer, Breast conserving therapy, Oncoplastic surgery, Volume replacement, Thoracodorsal adipofascial flap, Modification

## Abstract

**Background:**

The treatment of early breast cancer using breast conservation therapy (BCT) commonly ensures local control and acceptable cosmetic results. We report a useful technique including the use of a thoracodorsal adipofascial cutaneous flap for reconstructing defects in the outer quadrant area after partial mastectomy, which achieved excellent results.

**Methods:**

During the past 15 years, some modifications have been added to the original method at a rate of one modification every 2–5 years. We classified these modifications into the original method and four modified methods. Modification I: addition of a crescent-shaped dermis on the distant edge of the thoracodorsal adipofascial flap (TDAFF), Modification II: addition of a crescent-shaped dermis on the proximal edge of the TDAFF, Modification III: addition of inframammary formation plus Modification II, and Modification IV: change of a crescent-shaped dermis to a Benz-shaped (shaped like the Mercedes Benz logo) one plus Modification III. We compared the plastic period, postoperative complications, oncological results, and cosmetic results among the original and four modified groups.

**Results:**

The patient number was 26, 9, 15, 23, and 10 in the original and Modification I, II, III, and IV groups, respectively. The median observation period was 115, 92, 67, 51, and 32 months, respectively. Postoperative complications were seen in 5 (19%), 0, 2 (13%), 1 (5%), and 0 patients, respectively. Local recurrence was seen in 3 (12%), 0, 0, 0, and 0 patients, respectively. Distant recurrence was seen in 1 (4%), 1 (11%), 3 (20%), 0, and 0 patients, respectively. Cosmetic results evaluated as good–excellent were seen in 19 (73%), 5 (56%), 11 (73%), 19 (83%), and 10 (100%) patients, respectively.

**Conclusions:**

Oncoplastic surgery using an immediate volume replacement technique with a thoracodorsal adipofascial flap was improved by adding some modifications.

## Introduction

Breast conservation therapy (BCT) is well established as a treatment for breast cancer that provides local disease control with acceptable cosmetic results [1]. However, potential insufficient resection margins may increase the risk of local recurrence if too much attention is paid to cosmesis. Immediate reconstruction after BCT has thus become increasingly popular, even for early stage breast cancer [2, 3]. We previously reported our early experience of an oncoplastic technique including the use of a thoracodorsal adipofascial flap to repair partial defects in the upper outer quadrant area in Japanese women [4–6]. For a Japanese patient with relatively slim body, with non-ptotic breast, we should summarize our original technique to discuss how improved the original oncoplastic breast-conserving surgery using immediate volume replacement technique, and how modified them. We herein retrospectively review the modifications and their results performed in a single institution.

## Patients and Method

Between February 2004 and July 2018, 80 consecutive Japanese patients diagnosed with early breast cancer on the upper outer or lower outer quadrant area of non-ptotic breasts who underwent oncoplastic breast surgery combining breast-conserving surgery with immediate volume replacement using a thoracodorsal adipofascial flap in Kagoshima University Hospital were enrolled into this study. Indications for oncoplastic surgery combining partial mastectomy with immediate breast reshaping using a thoracodorsal adipofascial flap were as follows: (1) the patient had a non-ptotic breast with the nipple being centrally located from a frontal view, (2) the cancer lesion was restricted to the outer quadrant area, and (3) informed consent was preoperatively obtained after an explanation of the surgical procedures. Photographs were taken in four positions with the patient standing on floor marks: facing the camera with their arms down, facing the camera with their arms up, from the left side with their arms up, and from the right side with their arms up.

None of the patients who agreed to undergo this surgery received preoperative systemic chemo- or endocrine therapy. We added some modifications to the original method from experience at some points to make the operation procedure easier and improve both the oncological and cosmetic results. Every 2–5 years, a new modification was added. The original method was started in February 2004 (*n* = 26). Modification I: we added a crescent-shaped dermis on the distant edge of the flap in May 2009 (*n* = 9). Modification II: we added a crescent-shaped dermis on the proximal edge of the flap in August 2011 (*n* = 15). Modification III: we added plasticity of the inframammary line to Modification II in July 2013 (*n* = 23). Modification IV: we changed the shape of the dermis from crescent-shaped to Benz-shaped in Modification III in March 2016 (*n* = 10).

### Diagnosis and design

A preoperative study using mammography, ultrasonography (US), and computed tomography; a histological examination of a core needle biopsy sample; bone scintigraphy; and magnetic resonance imaging (MRI) were performed.

All patients were seen evaluated by the breast surgeon (M.H. and Y.K.) 1 day before surgery, so that she could plan the operation, make drawings, and explain the different surgical options, e.g., partial mastectomy without volume replacement, mastectomy following by immediate breast reconstruction.

**Fig. 1:**
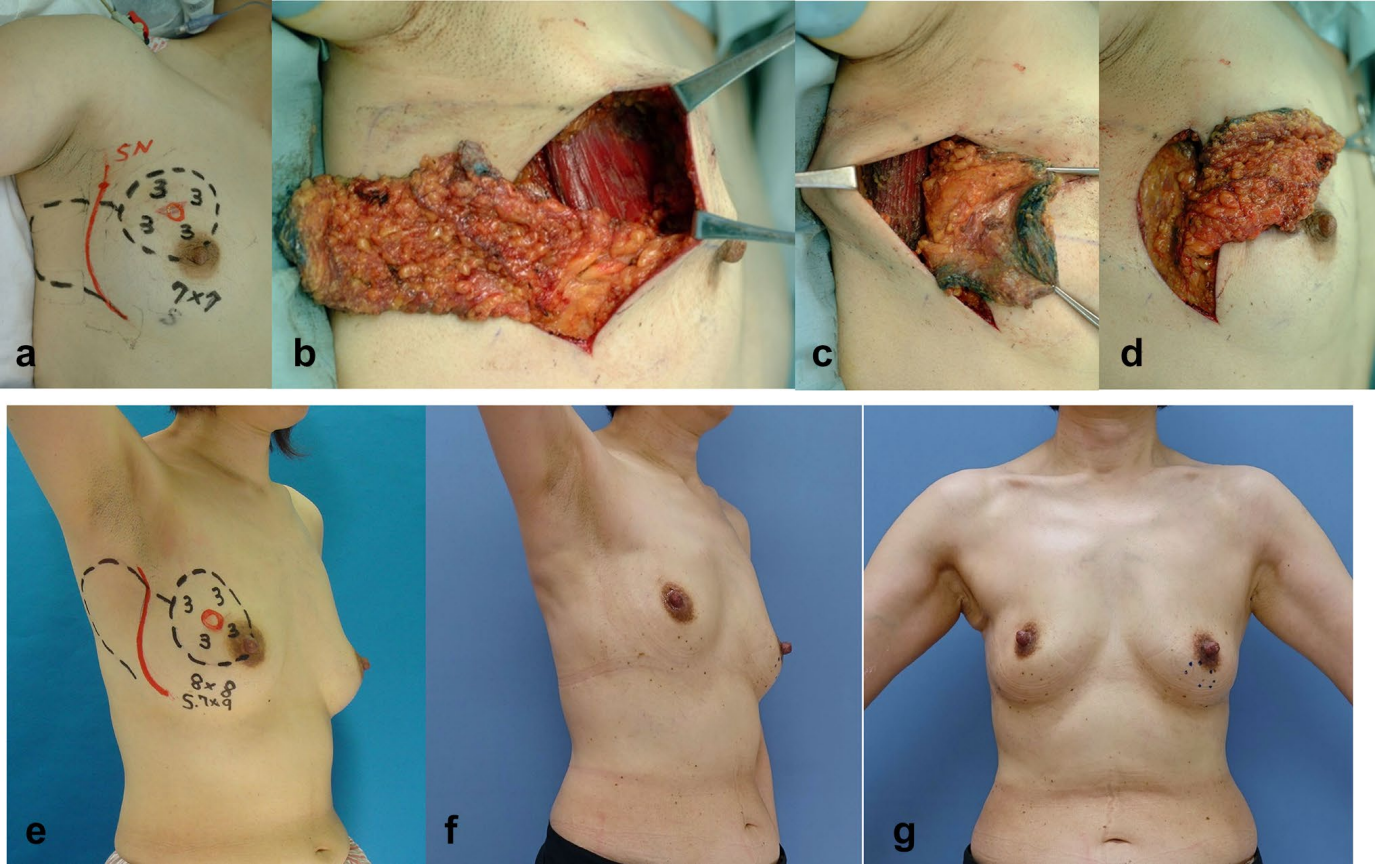
Original method, operation procedure, and gross findings. **a**, **e** Preoperative design, **b** column-shaped resection and raising of TDAFF, **c** muscular fascia was attached to the back surface of the TDAFF, **d** TDAFF was horizontally moved to fill the breast defect, and **f**, **g** 11 years postoperation

**Fig. 2:**
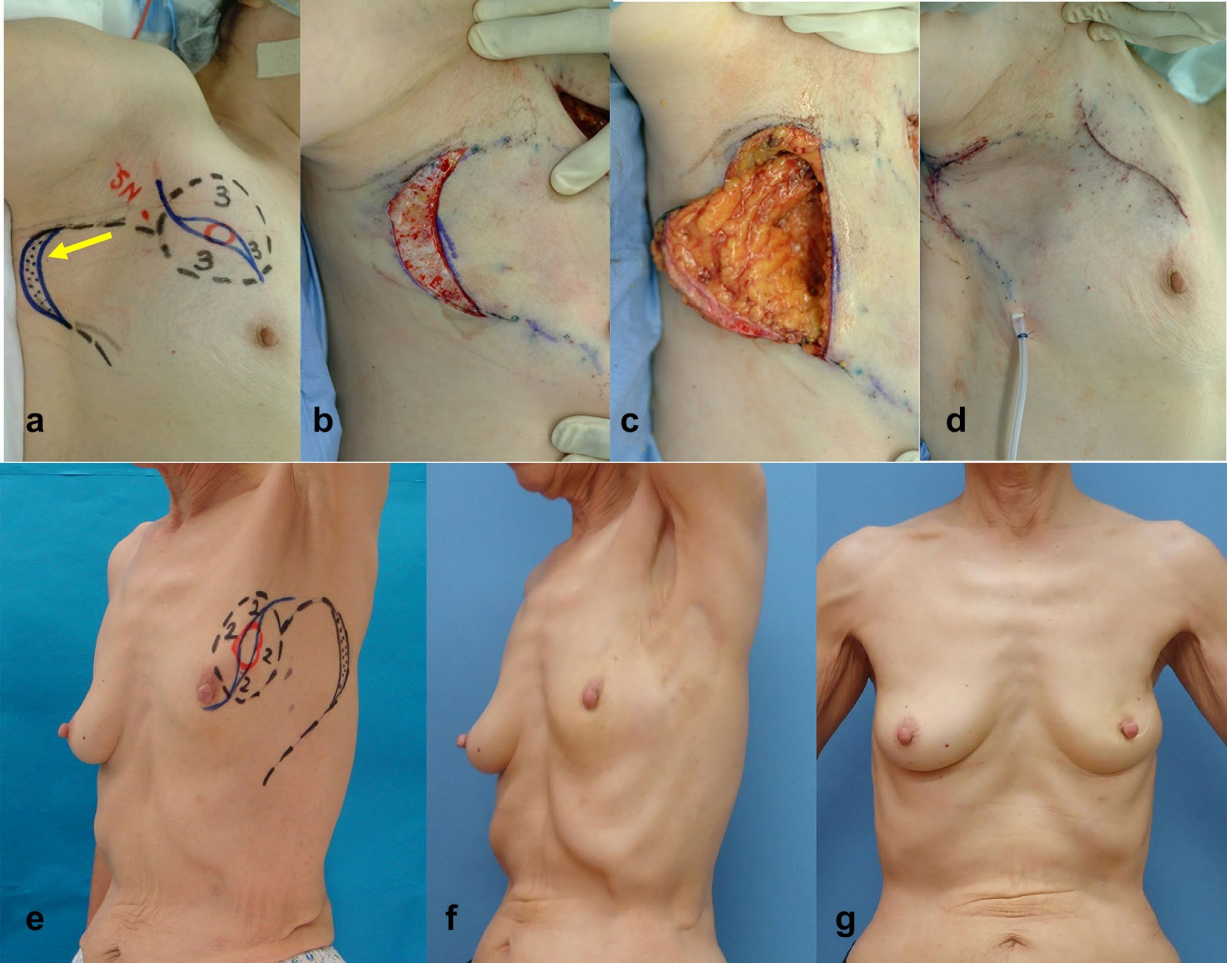
Modification I, operation procedure, and gross findings. **a**, **e** Preoperative design, **b** crescent-shaped area is de-epithelialized, **c** TDAFF with crescent-shaped dermis is raised via an incision along with the crescent on the proximal area of the flap. **d** TDAFF was horizontally moved to fill the breast defect, and **f**, **g** 4 years postoperation

**Fig. 3:**
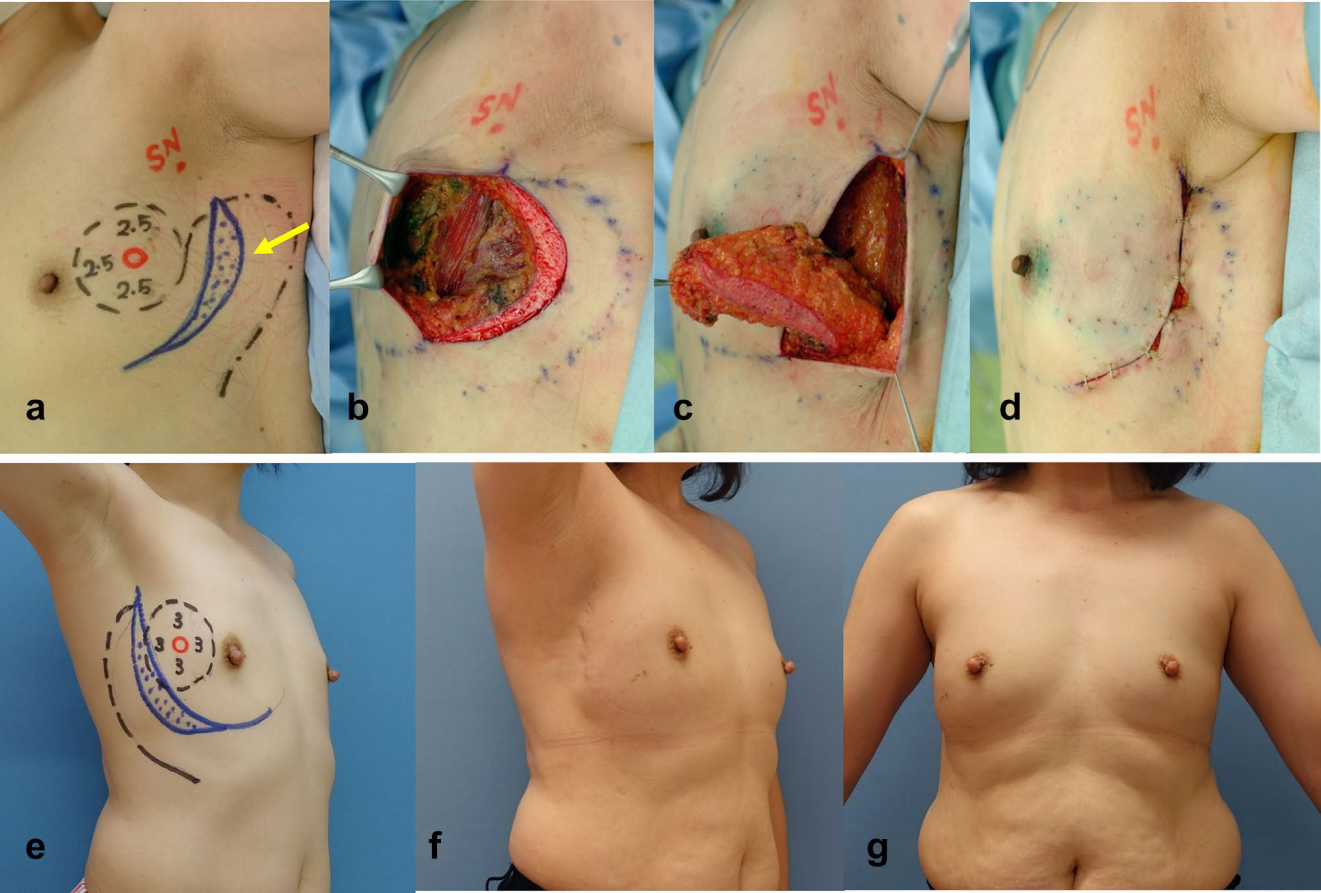
Modification II, operation procedure, and gross findings. **a**, **e**: Preoperative design, **b** crescent-shaped area is de-epithelialized, **c** TDAFF with crescent-shaped dermis is attached along with incision line, **d** TDAFF was horizontally moved to fill the breast defect, **f**, **g** 4 years postoperation

**Fig. 4:**
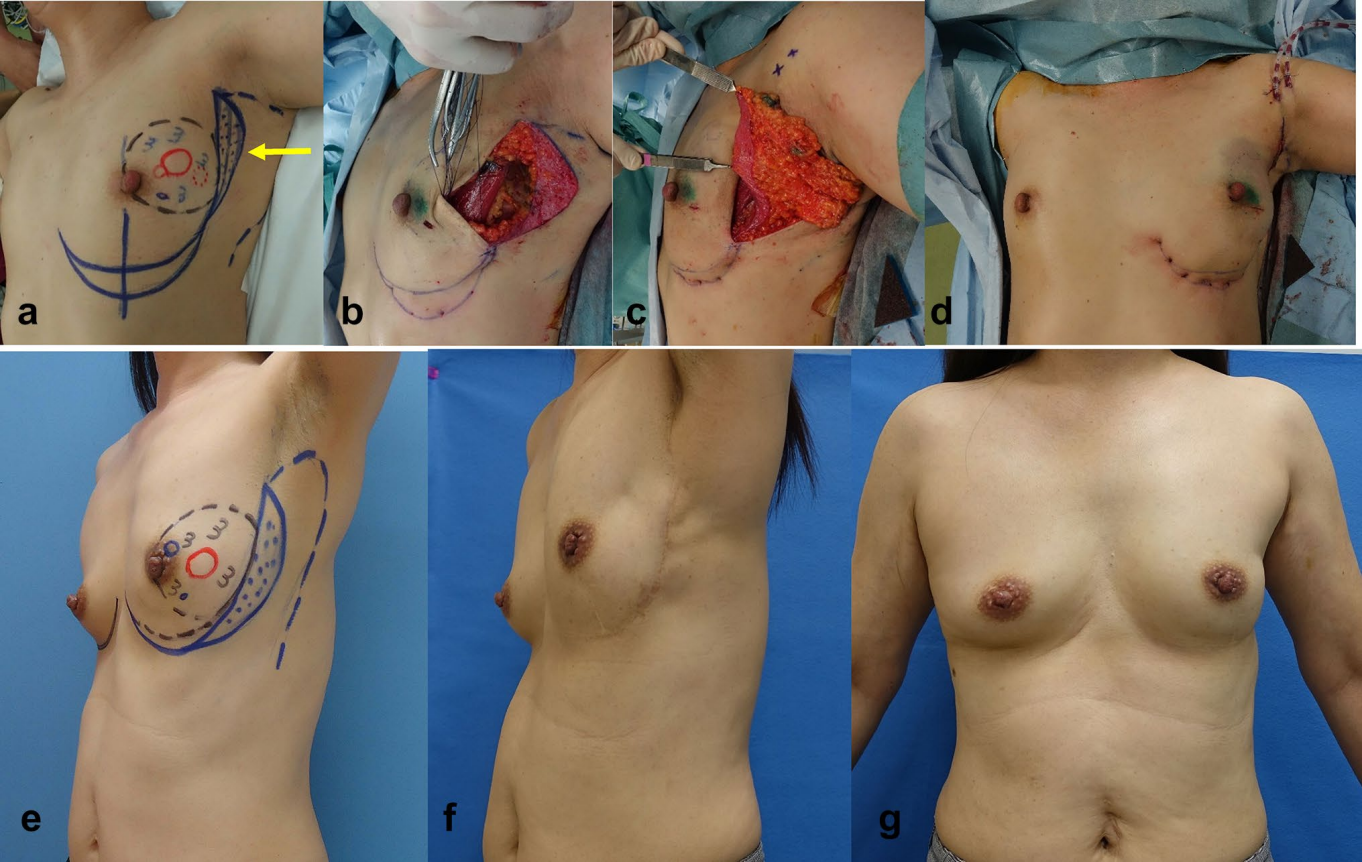
Modification III, operation procedure, and gross findings. **a**, **e** Preoperative design, **b** crescent-shaped area is de-epithelialized, **c** TDAFF with crescent-shaped dermis is attached along with incision line. Inframammary line is plastic using several stitches; **d** TDAFF was horizontally moved to fill the breast defect and **f**, **g** 4 years postoperation

**Fig. 5:**
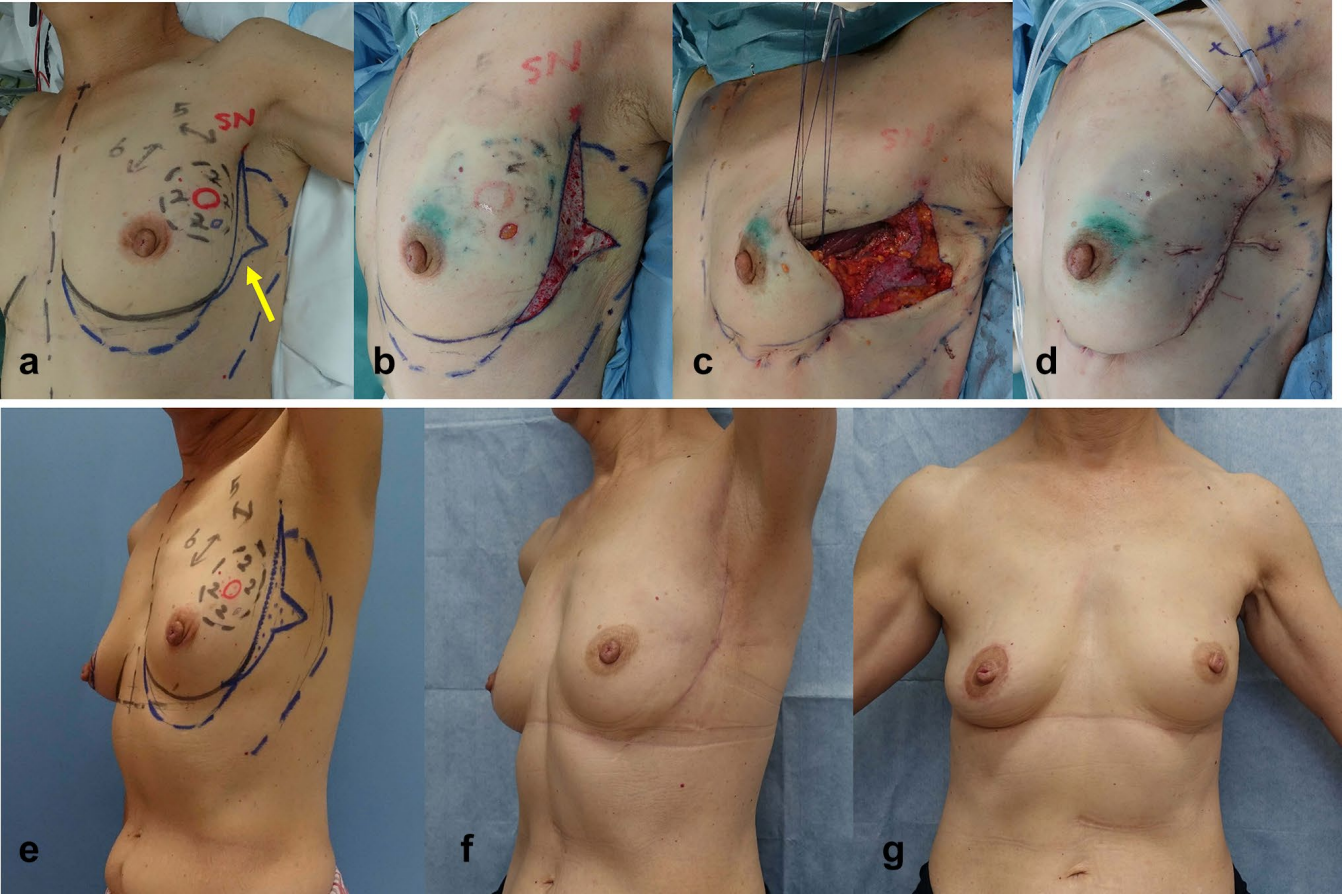
Modification IV, operation procedure, and gross findings. **a**, **e** Preoperative design, **b** Benz-shaped area is de-epithelialized, and **c** TDAFF with Benz-shaped dermis is attached along the incision line. Inframammary line is plastic using several stitches; **d** TDAFF was horizontally moved to fill the breast defect; **f**, **g** 2 years postoperation

Partial mastectomy was planned as follows: The preoperative, intraoperative, and postoperative findings of those groups in e, a-d, and f and g, in Figs. 1, 2, 3, 4, 5, respectively. The tumor area was diagnosed by mainly US and MRI. The tumor area was drawn using US with the patient in the supine position (red circle, in Figs. 1, 2, 3, 4, 5a and e). The surgical margin was drawn 2–3 cm apart from the red line (dotted black line, in Figs. 1, 2, 3, 4, 5a and e). We then drew a lateral edge of the breast with the patient in the standing position (Blue line, in Figs. 1, 3, 4, 5a and e). We also drew a new inframammary line on the 1.5–2.0 cm caudal site from the true one in Modification III and IV (Blue line, in Figs. 4 and 5a and e). Then, thoracodorsal adipofascial flap was marked (dotted black or blue line, in Figs. 1, 2, 3, 4, 5). Finally, de-epithethialized area was added along with the edge of the flap or incision line, Modification I and III–V, respectively. (Figs. 2 and 3, 4, 5a and e).

### Surgical procedure

Patients were seen by the breast surgeon (Y.K.) 1 day before surgery, so that she could plan the operation, make drawings, and explain different surgical options, e.g., other oncoplastic surgical techniques such as immediate volume displacement using a thoracodorsal adipofascial flap or a modified thoracodorsal cutaneous adipofascial flap as was routinely selected by previous patients at our institution [4, 5]. In all groups, partial mastectomy was performed by cylinder-shaped resection with complete resection of the fascia of the major pectoral muscle. Surgical margins were maintained at 2–3 cm. In all cases, several samples of the edges were examined pathologically to be shown as negative for cancer intraoperatively. The surgical procedure in each group is as below.

Original method: At the anterior axillary line, an incision line was planned with a lazy-s shape (Fig. 1). Via the incision, a c-shaped thoracodorsal adipofascial flap was raised with the fascia of the latissimus dorsi muscle [4].

Modification I: An incision was made to remove a leaf-shaped section of skin located just above the tumor (Fig. 2). Cylinder-shaped breast tissue was removed, and several margins were examined intraoperatively. A crescent-shaped dermis was de-epithelialized and attached to the distant edge of the TDAFF. For the entire circumference of the crescent, we incised toward the subcutaneous fatty tissue. Via this crescent incision, the TDAFF was harvested both from the surface and back surface of the flap at a point on the distant edge of it. The TDAFF with a crescent-shaped dermis was rotated and used to fill the deformity after partial mastectomy of the breast in the same manner as the original method [5].

Modification II: A crescent-shaped dermis was marked along with the incision line on the lateral curve of the breast. From the posterior curve of the crescent, we approached toward the edge of the flap with a shorter length than that of the original method (Fig. 3) [6].

Modification III: The design of the TDAFF was the same as Modification II. We add a procedure to make the inframammary line sharper as a modification. A new inframammary line was designed on an area 1.5–2.0 cm lower than the true inframammary one. We laid down 2–0 PDS® sutures in the subdermal layer and elevated them toward the cranial side; we then tied them without fixing to the chest wall. The sutured points were elevated toward the cranial side, resulting in the new inframammary line being clear [7]. It was resulted in maintaining the round shape of the lower part of the breast (Fig. 4).

Modification IV: We add one modification to Modification III by changing the dermis shape attached to the TDAFF from crescent-shaped to Benz-shaped, so that we could more easily reach the edge of the flap (Fig. 5).

### Axillary operations

Of the 80 patients, sentinel lymph-node biopsy was performed in 48 and axillary dissection in 32 (5 were converted from sentinel lymph-node biopsy to axillary dissection intraoperatively).

### Pathological findings

All specimens of the patients enrolled into this study were examined pathologically and found to be negative. No patients received delayed operation due to an involvement of the cancer.

### Postoperative therapy

Postoperative systemic therapy was selected according to the guidelines at that time. Postoperative radiation therapy was done for 5, 8, 15, 20, and 9 patients of the original and Modification I, II, III, and IV groups, respectively. Due to our institutional reasons, before 2010, postoperative radiation therapy was selectively done for patients with a pathological margin within 10 mm or with metastasis-positive lymph nodes.

### Cosmetic evaluation

A digital camera with a resolution of 18.2 megapixels was used with a blue panel as the background. Photographs were taken in several positions with the patients standing on floor marks: facing the camera with their arms down, facing the camera with their arms up, from the left side with their arms up, and from the right side with their arms up. In all patients except recurrent cases, we took photographs once or twice a year until the end of observation period. On patients who was diagnosed as recurrence, the latest time to be taken photographs was before recurrence. For patients without recurrence, the latest photographs were used for evaluation, so that the observation period and the evaluated period were almost the same. Images were recorded, printed out, and then cosmetically evaluated by two persons (Y.K. and M.H.) independently. For the evaluation of the softness of the breast we quoted the medical record when the patients visit outpatient clinic in our institution. Cosmetic assessment was based on the method by the Japanese Breast Cancer Society Sawai Group [8]. This assessment contains eight items: (1) breast size, (2) breast shape, (3) wound scar, (4) softness of the breast, (5) shape and size of nipple-areola, (6) color of nipple-areola, (7) level of nipple (difference of distance from suprasternal notch in bilateral nipples), and (8) lowest point of the breast (difference of bilateral breasts). The cosmetology was evaluated as excellent when the total score was 12 points, good when it was 9–11, fair when it was 5–8, and poor when it was 0–4. The cosmetology was evaluated as excellent when the total score was 12 points, good when it was 9–11, fair when it was 5–8, and poor when it was 0–4.

### Study protocol

This study protocol was approved by the ethics committee of Kagoshima University Graduate School of Medical and Dental Sciences and was consistent with the Declaration of Helsinki. All included patients provided their informed consent, as approved by the ethics committee.

## Results

The observation period (median) ranged from 32 to 115 months (Table 1). The average plastic period ranged from 46 to 59 min. Postoperative complications were seen in 5, 2, and 1 patient in the original method and Modifications II and III, respectively. They all were wound-healing delays with the incision line on the breast caused by superficial blood circulation disorder. Wide necrosis of the skin or bleeding was not seen. Of the 8 patients, two had fatty melting and fatty outflow and infection. We performed debridement of the scar of the disorder site as with the original method. No patient had complications associated with the donor site. The oncological findings are shown in Table 1. Local recurrence was seen in 3 patients with the original method: 2 in the remnant gland and 1 was needle-tract implantation. Distant recurrence was seen in 1, 1, and 3 patients in the original method and Modifications I and II, respectively.

**Table 1:** Clinical data and cosmetic results

	Original	Mod. I	Mod. II	Mod. III	Mod. IV
Patients number	26	9	15	22	10
Observation period, (median, months)	115	92	67	51	32
Plastic period (average, minutes)	59	46	55	51	56
Postoperative complication (wound-healing delay)^a^	5 (19%)	0	2 (13%)	1 (5%)	0
Recurrence					
Local recurrence	3 (12%)^b^	0 (0%)	0 (0%)	0 (0%)	0 (0%)
Distant recurrence	1 (4%)	1 (11%)	3 (20%)	0 (0%)	0 (0%)
Cosmetic results					
Good–excellent	20 (77%)	6 (67%)	12 (73%)	19 (86%)	10 (100%)
Fair	3 (13%)	2 (22%)	2 (13%)	2 (8%)	0 (0%)
Poor	2 (7%)	1 (11%)	1 (8%)	0 (0%)	0 (0%)
Not evaluated	1 (3%)	1 (11%)	0 (0%)	1 (5%)	0 (0%)
Cause of fair-, poor- cosmetic results					
Volume deficiency	3 (13%)	2 (22%)	3 (20%)	1 (5%)	0 (0%)
Nipple deviation	1 ( 3%)	1 (11%)	3 (20%)	1 (5%)	0 (0%)
Loss of IML	5 (19%)	0 (0%)	3 (20%)	1 (5%)	0 (0%)

^a^Blood circulation disorder along with incision line, all were cured without any surgical intervention

^b^Recurrent on the remnant gland (*n* = 2); recurrent due to needle-tract implantation (*n* = 1)

The cosmetic results are shown in Table 1 with 20 (77%), 6 (67%), 12 (73%), 19 (86%), and 10 (100%) patients evaluated as good-to-excellent, respectively. Three patients were not evaluated due to the patients’ wishes.

**Fig. 6:**
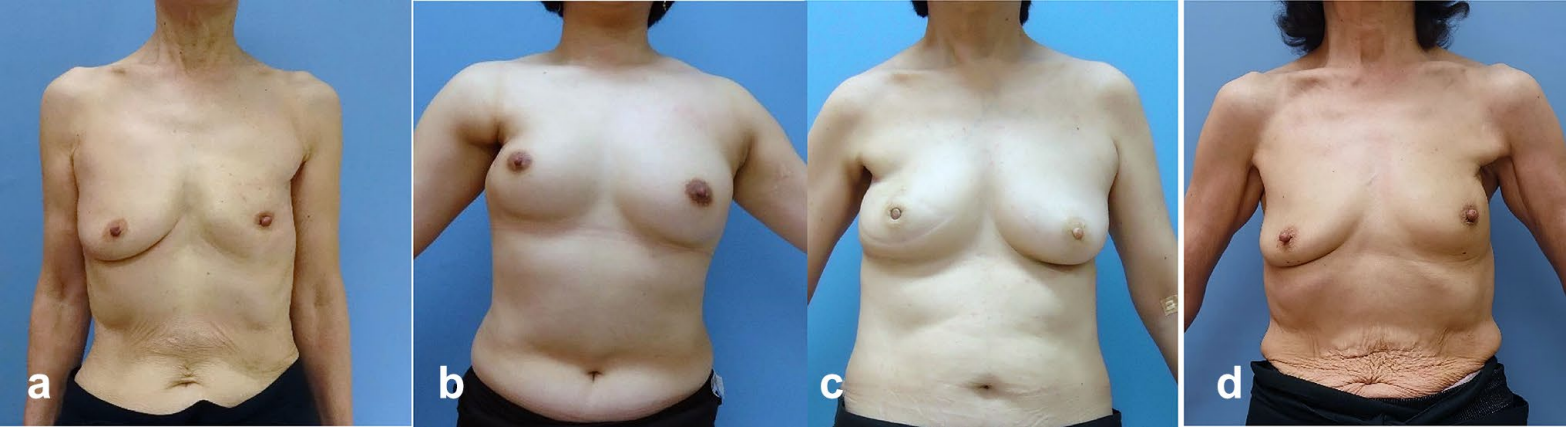
Gross findings of fair-poor cosmetic results in four groups: **a** original method, fair, volume deficiency, and loss of IML. **b** Modification I, poor, nipple deviation, volume deficiency. **c** Modification II, fair, volume deficiency, and loss of IML. **d** Modification III, poor, nipple deviation, and loss of IML

We picked up the reason which is thought to relate to poor cosmetic results in all patients with the cosmetic evaluation as fair or poor. There are 5, 3, 3, 2, and 0 patients with fair or poor evaluation in original Modification I, II, III, and IV groups, respectively. Loss of the inframammary line was detected in 5, 3, and 1 patients in Original, Modification II, and III, respectively (Fig. 6).

Out of those 13 patients with fair of poor cosmetic results, 2 patients lost IML, 8 patients received axillary lymph adenectomy, 2 had both of them (loss of IML, and axillary lymph adenectomy), and one experienced fatty melting postoperatively. On the other hand, in the patients with good-to-excellent cosmetic results, 20 (30%) received axillary lymph-node dissection rather than sentinel lymph-node biopsy. The causes of fair-to-poor cosmetic results were volume deficiency and nipple deviation. Two patients out of 5, 2 patients out of 3, and 1 patient out of 1 in Original, Modification II, and III, whose IML was disappear were evaluated as fair-to-poor in cosmetic evaluation, respectively. In modification III and IV, all 31 patients except one have kept clear IML. There was no significant relationship between postoperative cosmetic evaluation and oncological data, clinical factor such as patients’ age, body mass index, and menopausal status.

## Discussion

In the 1980s, BCT rapidly became a first-line procedure for early stage breast cancer as it promoted local breast disease control and had an acceptable cosmetic impact [1, 2]. Early research suggested that breast tissue conservation or restitution of the breast may mitigate the negative effects of breast cancer on the sexual well-being of females, and the cosmetic results exerted a marked effect on the subsequent psychological outcomes [3, 9].

The main aims of BCT are both locoregional control and survival, as well as a good cosmetic outcome. Oncoplastic techniques, which combine the concepts of oncologic and plastic surgery, are becoming more common, especially in Western countries [10–12]. Volume replacement is one of the main types of OBS, along with volume displacement [12]. According to a comparison of the local control between OBS and conventional BCT, the positive-margin rate of cancer was significantly lower in the OBS groups. The introduction of OBS was to reduce the rate of delayed operation due to positive margins [13, 14]. We introduced OBS into Japanese patients diagnosed with early breast cancer suitable for BCT in 2003. We selected volume replacement or volume displacement according to whether their breast was ptotic or non-ptotic. Especially in the case of volume replacement, we select adequate extra breast tissue to repair the breast defect immediately at the time of operation. From these experiences, we are aware that immediate volume replacement using extra breast tissue quadrant by quadrant is a useful technique for patients with small, non-ptotic breasts [15–17].

We have used oncoplastic surgery for patients with early breast cancer on the outer area of the breast using a TDAFF from 2005 until now. During this period, some modifications were added to the original immediate volume replacement technique based on experience. Modification I was useful for patients whose lesions necessitated the removal of skin just above the tumor. The distance from the incision on the breast to the distant edge of the TDAFF was longer than that of the original method. We approached via an additional incision which is located on the distant edge of the flap. The attached epidermis may improve blood flow, revascularization, and flap survival compared with flaps created in the original manner [18]. In Modifications II, III, and IV, we placed the epithelialized skin along the lateral curve of the breast. The formed operation scar was inconspicuous from the anterior view. The length necessary to reach the distant edge of the flap was shorter in the width of the crescent-shaped dermis. Via a distant curve of the crescent, we started to harvest the flap and reached the edge of it easily. Based on our previous research, the attached dermis supported blood flow well and made the flap volume larger [6]. During the skin closing procedure, some considerations were necessary, because the two lengths of the anterior and posterior curves of the crescent area were different. Puckering was formed just after the operation; however, it disappeared within one postoperative year. In Modifications III and IV, we tried to make the inframammary line sharper by adding subdermal stitches. Until now, it was thought to be successful; however, it may need longer follow-up observation. In Modification IV, we changed the shape of the attached dermis from crescent-shape to Benz-shape. Using this modification, it became not only easier to reach the distant edge of the flap but also enabled the flap volume to be larger. A horizontal scar was formed after skin closure; however, it was short and inconspicuous from the anterior view.

We have only two cases with fatty melting out of 80 procedures. This is one of the most important point of partial mastectomy with immediate volume replacement. As we described in the former study, our procedure is characterized by the blood flow from the backside of the flap, via perforators from chest wall [6]. To keep them, we avoid undermining the flap from serratus anterior muscle as well as possible. Secondary to minimize damages of the flap, we treated the flap carefully very much. It is important to avoid catching it by the forceps; instead, we used the special forceps with hook (Fig. 4c).

These techniques are easily performed by breast surgeons without any change of the patients’ body position intraoperatively in comparison to other techniques such as volume replacement using a latissimus dorsi muscle flap or a perforator flap [19, 20].

In this study, the cosmetic results were worse in patients with axillary lymph-node dissection. When we removed the cancer lesion on the outer upper quadrant area, the partial defect became larger using an axillary approach following partial mastectomy of the breast. We found that when we rotated, filled, and fixed the TDAFF, the breast defect moved toward the cranial side of the planned position. The skin covering the pectoral major muscle or axillary area may gradually shrink. This deviation or volume loss was seen in patients with the original method and Modifications I, II, and III, but not in Modification IV. It is thought that these experiences helped us to reduce negative postoperative cosmetic changes at the time of operation. Further consideration and evaluation are needed.

From this study, we consider that Modifications I and IV are useful. Although the cosmetic results with Modification I were worse, it is useful for patients in whom the skin on the tumor should be removed together for oncological reason. Also, it is useful for patients with extremely thin breasts. We are aware that longer periods of observation and physical and cosmetic assessments for Modification IV are needed. After limited observation in a small number of patients, we found that this procedure provided excellent cosmetic results when performed by a breast surgeon. Oncoplastic surgery, combining partial mastectomy and immediate volume displacement using a modified TDAFF with a Benz-shaped dermis at the time of BCT can be performed easily and safely and produces good cosmetic results in patients who are not indicated for reduction-type oncoplastic surgery.

We aware that this study is lacking patients’ satisfaction and complications by themselves. Also, the cosmetic evaluations were done by surgeons but not medical staffs such as nurses. They would be resulted in making the value of this study be lower; however, we would like to report that it has been performed in the one institution under the distinctive oncological indication and patient’s selection.

We also recognized the other point that we should solve. The postoperative period from surgery and cosmetic evaluation are different in each group. This study is postoperative review in which some modifications are added to original volume replacement surgery for Japanese patients with a breast cancer on the upper outer quadrant are with non-ptotic breast in one institution. From our former experience for oncoplastic breast-conserving surgery using volume replacement techniques, the cosmetic results have been maintained for a several years without major changed; however, it is necessary to unify the time of cosmetic evaluation. We have a plan to solve it in the near future.

From our experience, the necessity of modifications occurred due to different experiences, which resulted in making the operation procedure easier and cosmetic results better. To solve the lacks of this study, further study are necessary.

## Conclusion

Continuous experiences of modifications to immediate volume replacement using a thoracodorsal adipofascial flap for patients with early breast cancer on the outer area of the breast gave improvement of the operation procedures and excellent cosmetic results.
